# Endocrine resistance in HR+/HER2− breast cancer: molecular mechanisms and emerging therapeutic strategies

**DOI:** 10.3389/fonc.2026.1983430

**Published:** 2026-10-16

**Authors:** Tong Shen, Hui Li, Xubin Zhang, Qin Zhou

**Affiliations:** 1 School of Medicine, Jiangsu University, Zhenjiang, China; 2 Department of Breast Surgery, Affiliated Kunshan Hospital of Jiangsu University, Suzhou, China

**Keywords:** circulating tumor DNA (ctDNA), endocrine resistance, ESR1 mutation, HR-positive/HER2-negative breast cancer, oral SERD, PI3K/Akt/mTOR pathway, precision therapy, PROTACs

## Abstract

Hormone receptor–positive/human epidermal growth factor receptor 2–negative (HR+/HER2−) breast cancer accounts for the majority of breast cancers and remains highly dependent on endocrine therapy across both early and advanced disease settings. Although the incorporation of CDK4/6 inhibitors has substantially improved clinical outcomes, endocrine resistance, whether primary or secondary, ultimately limits durable disease control. Resistance arises through heterogeneous and evolving mechanisms, including ESR1 mutations and fusions, ER transcriptional reprogramming, activation of PI3K/AKT/mTOR and other bypass pathways, cell-cycle escape, epigenetic remodeling, and metabolic adaptation. These molecular insights are increasingly being translated into biomarker-guided therapeutic strategies, including next-generation oral selective estrogen receptor degraders, PROTAC ER degraders, and PI3K/AKT/mTOR inhibitors; antibody–drug conjugates are considered separately as antigen-directed options after endocrine resistance. Importantly, circulating tumor DNA monitoring of emerging ESR1 mutations provides an opportunity to identify molecular progression before radiographic progression and to support earlier treatment adaptation. This narrative review integrates recent mechanistic and clinical evidence on endocrine resistance in HR+/HER2− breast cancer, with emphasis on how resistance mechanisms and biomarkers can inform drug selection, treatment sequencing, and dynamic disease monitoring. A mechanism-guided approach may therefore help move clinical management from empiric treatment switching toward more individualized, biomarker-driven, whole-course care.

## 1 Introduction

Breast cancer is the most commonly diagnosed malignancy among women worldwide (1), and approximately 70% of cases are hormone receptor–positive (HR+) and human epidermal growth factor receptor 2–negative (HER2−) (2). Extensive evidence has shown that estrogen receptor alpha (ERα) is a key biomarker of this subtype and a transcription factor that drives tumor growth. Endocrine therapy (ET), which acts by blocking the binding of estrogen to its receptor or by reducing systemic estrogen levels, has long been the standard treatment for these patients (2–5). In recent years, the combination of cyclin-dependent kinase 4/6 (CDK4/6) inhibitors with endocrine therapy has significantly prolonged progression-free survival (PFS) and overall survival (OS) in patients with advanced disease, thereby establishing a new standard for first-line treatment. However, with long-term drug exposure, tumor cells may acquire resistance through genomic evolution and transcriptional adaptation, ultimately leading to disease progression. Therefore, a deeper understanding of the molecular mechanisms underlying resistance is of great clinical importance for identifying high-risk populations, guiding therapeutic decision-making, and developing novel targeted agents (6). Clinically, endocrine resistance can be classified as primary or secondary. According to the updated ABC6/7 consensus definitions, primary endocrine resistance refers to relapse during the first 2 years of adjuvant endocrine therapy or progressive disease within the first 6 months of first-line endocrine therapy–based treatment for advanced breast cancer while receiving endocrine therapy. Secondary endocrine resistance encompasses relapse after the first 2 years of adjuvant endocrine therapy, progression after at least 6 months of first-line endocrine therapy–based treatment for advanced disease, progression during any duration of second or subsequent endocrine therapy–based lines, and known ESR1 mutation. These definitions are mainly intended to standardize clinical-trial populations; in clinical practice, endocrine sensitivity and resistance are better viewed as a continuum and should be interpreted together with prior treatment exposure, disease tempo, and molecular biomarkers (7).

This article is intended as a narrative review. We prioritized recent mechanistic studies, pivotal clinical trials, consensus guidance, and regulatory reports relevant to endocrine resistance in HR+/HER2− breast cancer. It does not use a prespecified systematic-review protocol, formal risk-of-bias assessment, or PRISMA workflow; the evidence is therefore synthesized qualitatively.

## 2 Research progress on the molecular mechanisms of endocrine therapy resistance

### 2.1 Alterations in the estrogen receptor gene (ESR1) and transcriptional reprogramming

Estrogen receptor alpha (ERα) is encoded by the ESR1 gene, and structural alterations in this receptor represent one of the most direct and well-defined mechanisms of acquired resistance. ESR1 mutations are an important cause of acquired endocrine resistance (8). These mutations are extremely rare in untreated primary breast cancer, but their frequency increases markedly to approximately 20%–40% in patients with metastatic disease who have received aromatase inhibitor (AI) therapy (8–10). Studies have shown that these mutations are mainly clustered in the ligand-binding domain (LBD), with Y537S and D538G being the most common mutation sites (4). Missense mutations at these sites alter the spatial conformation of the ER protein, enabling it to recruit coactivators and maintain sustained transcriptional activity even in the absence of estrogen ligand binding. This ligand-independent activation renders AI agents, which act by lowering estrogen levels, ineffective. At the same time, it also reduces the binding affinity of the first-generation SERD fulvestrant for the receptor, thereby leading to partial cross-resistance.

In addition, recent whole-transcriptome sequencing studies have revealed that ESR1 gene fusions represent another important mechanism of acquired endocrine resistance (11). Whole-transcriptome sequencing analyses have identified fusion genes such as ESR1–YAP1 and ESR1–CCDC170 in endocrine-resistant metastatic lesions (11, 12). Further investigation of endocrine-resistant metastases has shown that, following ESR1–YAP1 fusion, the transcriptional activation domain of YAP1 replaces the ligand-binding domain of ER, thereby enabling estrogen-independent cell growth and conferring resistance to anti-estrogen therapy. By contrast, ESR1–CCDC170 forms a complex with HER2/HER3/SRC, activating the SRC–PI3K–AKT signaling pathway and subsequently driving cellular resistance. Knockdown of this fusion gene reduces HER2/HER3 expression and restores sensitivity to endocrine therapy (13).

However, resistance does not arise solely from structural genetic alterations (14, 15). Resistant cells often exhibit marked transcriptional reprogramming, in which epigenetic modifications enhance the binding of ER to specific chromatin sites and sustain transcriptional programs required for tumor survival (16, 17). This phenomenon of “transcriptional dependency” increases the complexity of treatment and suggests that a single-target strategy may be insufficient to fully suppress the growth of resistant clones.

### 2.2 Crosstalk and compensatory activation of growth factor receptor signaling pathways

When the ER signaling pathway is pharmacologically inhibited, tumor cells do not necessarily stop growing (18). In many cases, they shift toward other growth factor–related pathways to sustain proliferation and survival (19). This interaction between signaling pathways is commonly referred to as crosstalk, and it represents one of the important mechanisms underlying resistance after endocrine therapy. Among these pathways, the PI3K/AKT/mTOR pathway has received particular attention (20). Clinical studies have shown that approximately 40% of patients with HR+/HER2− breast cancer harbor activating PIK3CA mutations (21). These mutations can promote tumor cell proliferation and may also induce ligand-independent phosphorylation of ER through downstream kinases such as S6K1, thereby enhancing ER transcriptional activity even in the absence of estrogen stimulation (22). On the other hand, when endocrine therapy suppresses ER signaling, the negative feedback originally maintained by ER is weakened. As a result, AKT phosphorylation increases and further affects receptor tyrosine kinase signaling mediated by receptors such as HER3 and IGF1R. Over time, tumor cells may develop a survival pattern that depends increasingly on bypass signaling pathways, which is one of the major causes of acquired resistance (23).

The role of HER2/HER3 signaling in endocrine resistance also deserves attention (24). This mechanistic concept refers to pathway activity and receptor crosstalk rather than to the clinical HER2-low category itself. Even without HER2 amplification, ligand-driven HER2/HER3 heterodimerization may activate SRC/PI3K/AKT signaling and reshape ER transcription through the HER2/HER3–HDAC2–FOXA1 axis. HDAC2-mediated FOXA1 deacetylation alters FOXA1 genomic binding and redirects ER toward resistance-, invasion-, and metastasis-associated loci (25–27). Accordingly, HER2-low, HER2-ultralow, or IHC 0 classification should not be interpreted as a direct mechanism of endocrine resistance; these are treatment-selection descriptors for antibody–drug conjugates, whereas HER2/HER3 pathway activation is the relevant mechanistic feature.

### 2.3 Dysregulation of cell-cycle control and resistance to CDK4/6 inhibitors

With the widespread clinical use of CDK4/6 inhibitors, resistance to these agents has gradually attracted increasing attention (28, 29). Their antitumor activity depends on an intact and functional Rb protein (30, 31). Under normal conditions, CDK4/6 inhibitors block cell-cycle progression from the G1 phase to the S phase, thereby restricting tumor cell proliferation (32). However, when the RB1 gene undergoes loss-of-function mutations or gene deletion, this critical step in drug action is disrupted. After loss of Rb function, E2F transcription factors are no longer effectively suppressed, allowing the cell cycle to continue progressing. At the same time, tumor cells become less dependent on the CDK4/6–Cyclin D1 complex, making it difficult for CDK4/6 inhibitors to exert their original cell-cycle blocking effect. Current evidence suggests that RB1 inactivation is not highly frequent in HR+/HER2− metastatic breast cancer, but its impact on therapeutic efficacy is relatively well defined. Among patients treated with palbociclib plus fulvestrant, those with RB1 loss have a markedly shorter median progression-free survival, suggesting that this alteration may represent an important mechanism of acquired resistance to CDK4/6 inhibitors (33).

In addition to RB1 alterations, abnormalities in downstream cell-cycle regulators may also affect the efficacy of CDK4/6 inhibitors (34, 35). Amplification of Cyclin E1 (CCNE1) or overexpression of CDK2 can promote the formation of the Cyclin E–CDK2 complex, allowing tumor cells to rely more heavily on this complex to drive cell-cycle progression (36). Recent single-cell sequencing studies have also suggested that resistance is not solely attributable to intrinsic changes within tumor cells (37). Under the selective pressure of CDK4/6 inhibitor treatment, immunosuppressive cell populations within the tumor microenvironment may increase, thereby facilitating the survival of resistant cells (38). In some tumor cells, loss of FAT1 may also occur, leading to inactivation of the Hippo pathway (39). The major mechanisms of endocrine therapy resistance in HR+/HER2− breast cancer are summarized in **Figure 1**. Representative resistance mechanisms, associated biomarkers, and corresponding therapeutic strategies are summarized in **Table 1**. These adaptive changes in the microenvironment and signaling pathways promote the expansion and survival of resistant clones.

**Figure 1:**
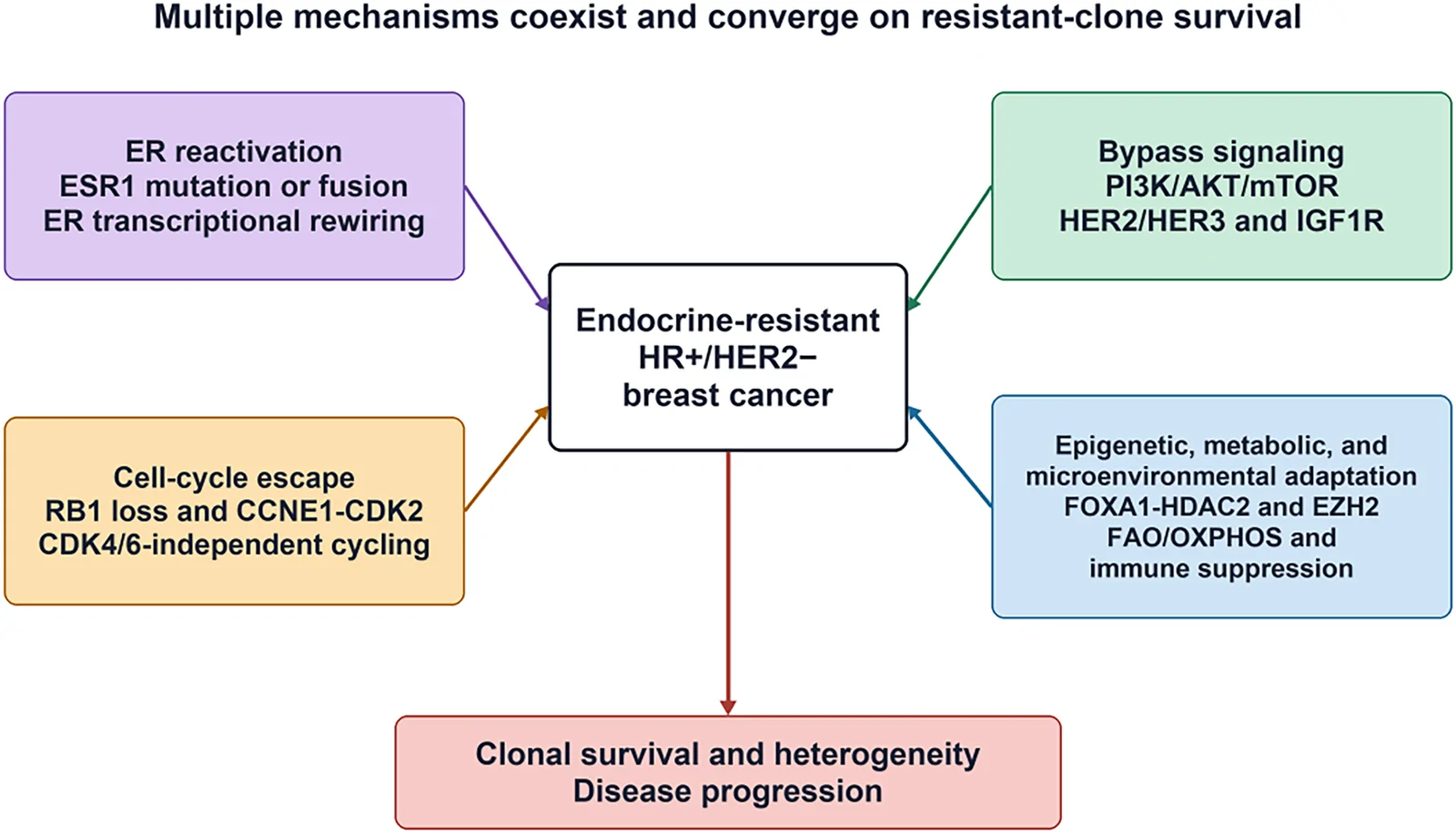
Major mechanisms of endocrine therapy resistance in HR+/HER2− breast cancer.

**Table 1:** Representative mechanisms, biomarkers, and therapeutic strategies for endocrine-resistant HR+/HER2− breast cancer.

Mechanism	Biomarker/feature	Therapeutic strategy	Representative agents
ER alteration	ESR1 mutation/fusion	ER targeting	Elacestrant; vepdegestrant
Bypass signaling	PIK3CA/AKT1/PTEN	PI3K/AKT/mTOR blockade	Inavolisib; capivasertib; everolimus
Cell-cycle escape	RB1 loss; CCNE1/CDK2	Cell-cycle targeting	CDK4/6- or CDK2-directed strategies
Epigenetic remodeling	HDAC2/FOXA1; EZH2	Epigenetic therapy	HDAC or EZH2 inhibitors
Metabolic adaptation	FAO/OXPHOS dependence	Metabolic targeting	Investigational inhibitors

## 3 Therapeutic strategies to overcome resistance and advances in drug development

### 3.1 Next-generation ER-targeted agents: oral SERDs, PROTACs, and CERANs

Given that ESR1 mutations represent a core mechanism of endocrine resistance, the development of novel agents capable of effectively binding mutant receptors while offering improved pharmacokinetic properties has become a major therapeutic direction. Compared with fulvestrant, next-generation oral selective estrogen receptor degraders (SERDs) have advantages in route of administration and bioavailability, making them more suitable for long-term treatment (40–42). Elacestrant is currently the first approved oral SERD. The phase III EMERALD trial showed that, among patients who had previously received CDK4/6 inhibitors and harbored ESR1 mutations, elacestrant monotherapy significantly prolonged progression-free survival (PFS) compared with standard endocrine therapy. Notably, patients with a longer duration of prior CDK4/6 inhibitor treatment appeared to derive greater benefit, which may indicate that their tumors retained a certain degree of endocrine sensitivity (43). Another oral SERD, camizestrant, also demonstrated favorable efficacy in the SERENA-2 study (44). Its therapeutic effect was superior to that of fulvestrant, regardless of the presence or absence of ESR1 mutations. With the development of detection technologies, real-time monitoring of ESR1 mutations and fusions has become increasingly feasible. Liquid biopsy studies have shown that, in previously endocrine-treated HR+/HER2− metastatic breast cancer, the detection rate of ESR1 mutations may reach 20%–50%, and these mutations are often associated with poorer responses to aromatase inhibitors and shorter PFS (45, 46). The randomized PADA-1 trial used circulating tumor DNA (ctDNA) to track ESR1 mutation levels. Once an increase in mutation levels was detected, treatment was switched from “aromatase inhibitor plus palbociclib” to “fulvestrant plus palbociclib.” (47, 48) This strategy significantly prolonged median PFS, further supporting the mechanism by which ESR1 mutations and fusions contribute to tumor resistance, as well as the clinical value of ctDNA monitoring. Taken together, these findings suggest that ctDNA testing is not only useful for detecting ESR1 mutations, but, more importantly, may help clinicians adjust treatment before radiographic or clinical disease progression occurs (47–49). The phase III SERENA-6 trial further demonstrated the potential of biomarker-guided early intervention with camizestrant. In this trial, patients receiving first-line aromatase inhibitor plus CDK4/6 inhibitor therapy were serially monitored for ESR1 mutations in circulating tumor DNA (ctDNA). Patients with newly detected ESR1 mutations but without radiological disease progression were switched from the aromatase inhibitor to camizestrant while continuing the same CDK4/6 inhibitor. With longer follow-up, the extended analysis showed a median PFS of 16.8 versus 9.2 months (HR, 0.45; 95% CI, 0.34–0.59) and a median second progression-free survival (PFS2) of 25.7 versus 19.1 months (HR, 0.63; 95% CI, 0.46–0.86), further supporting ctDNA-guided treatment modification before radiological progression (50).

Assay performance should be interpreted by platform rather than as a fixed property of ctDNA testing. Multiplex droplet digital PCR (ddPCR) is well suited to serial monitoring of predefined ESR1 hotspots and has reported analytical limits of detection of 0.07%-0.19% mutant allele fraction, but it will miss variants outside the probe set. Targeted next-generation sequencing (NGS) provides broader coverage of ESR1 and concurrent resistance alterations; however, sensitivity depends on read depth, molecular error suppression, DNA input, and variant allele fraction. In one proof-of-concept dPCR-SEQ comparison, sensitivity was 79% and specificity was 100% relative to conventional dPCR, with discordance confined to mutations below the 1.6% assay threshold, underscoring that reported values are platform-specific rather than directly interchangeable (51, 52). Clinically validated plasma assays generally achieve high specificity, whereas false-negative results remain possible when the ctDNA fraction is low, tumor shedding is limited, or the alteration is a fusion or copy-number event. Therefore, a negative or non-informative plasma result should prompt repeat testing and/or tissue-based analysis when clinical suspicion is high; RNA-capable tissue assays may be needed for ESR1 fusions (53). A practical workflow is shown in **Figure 2**.

**Figure 2:**
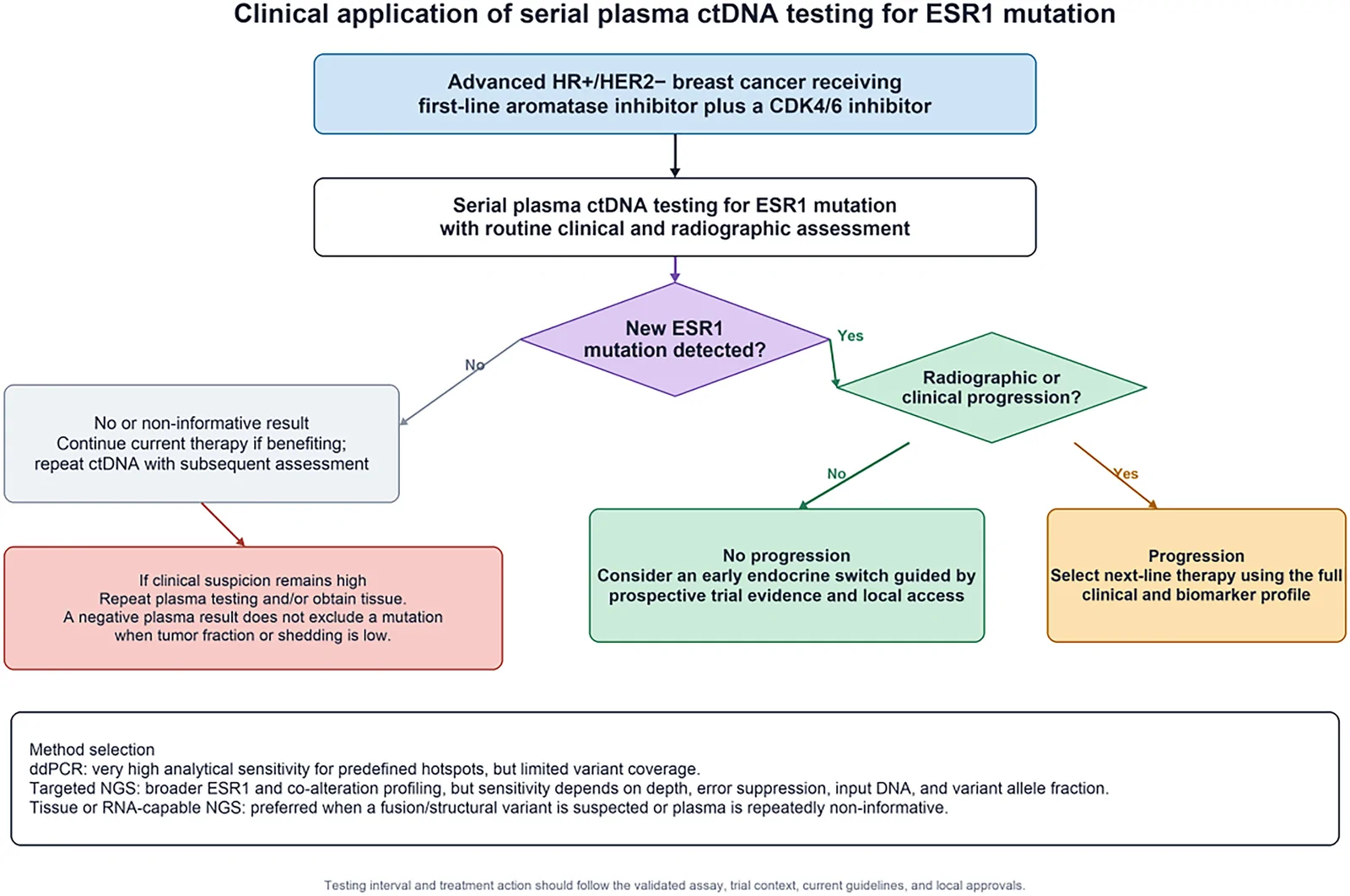
Clinical application of serial ctDNA monitoring for ESR1 mutations in advanced HR+/HER2− breast cancer. A negative or non-informative plasma result should be interpreted in the context of tumor fraction, disease burden, and assay coverage and may require repeat plasma or tissue testing.

In addition to SERDs, proteolysis-targeting chimeras (PROTACs) have emerged in recent years as a new direction in ER-targeted therapy (54, 55). Represented by vepdegestrant (ARV-471), PROTACs harness the cell’s endogenous ubiquitin–proteasome system to directly degrade ER (56). Studies have shown that ARV-471 can markedly induce ER degradation at relatively low concentrations and is active against both wild-type and mutant ER, with some findings suggesting that ER degradation may exceed 90%. Moreover, when combined with CDK4/6 inhibitors or inhibitors of the PI3K/mTOR pathway, ARV-471 has shown a degree of synergistic activity. The phase III VERITAC-2 trial compared vepdegestrant with fulvestrant in patients with ER+/HER2− advanced breast cancer previously treated with a CDK4/6 inhibitor and endocrine therapy (57). In the ESR1-mutated population, vepdegestrant significantly prolonged median PFS compared with fulvestrant (5.0 vs. 2.1 months; HR, 0.58; 95% CI, 0.43–0.78; P < 0.001). However, a statistically significant PFS benefit was not observed in the overall population (3.8 vs. 3.6 months; HR, 0.83; 95% CI, 0.69–1.01; P = 0.07). These findings suggest that ESR1 mutation status may help identify patients who are more likely to benefit from vepdegestrant (58).

Imlunestrant, another next-generation oral SERD, has demonstrated clinical efficacy in advanced HR+/HER2− breast cancer. In the phase III EMBER-3 trial, imlunestrant significantly prolonged PFS compared with standard endocrine therapy in patients with ESR1 mutations (median PFS, 5.5 vs. 3.8 months), although no significant PFS benefit was observed with imlunestrant monotherapy in the overall population (59). Moreover, imlunestrant combined with abemaciclib significantly improved PFS compared with imlunestrant alone, regardless of ESR1 mutation status. In the updated analysis with longer follow-up, median PFS was 10.9 versus 5.5 months with imlunestrant plus abemaciclib versus imlunestrant alone (HR, 0.59; 95% CI, 0.47–0.74). In the ESR1-mutated population, median OS was 34.5 versus 23.1 months with imlunestrant versus standard endocrine therapy (HR, 0.60; 95% CI, 0.43–0.86), although the prespecified boundary for statistical significance was not reached (60). Based on the EMBER-3 results, the FDA approved imlunestrant on September 25, 2025, for adults with ER+/HER2−, ESR1-mutated advanced or metastatic breast cancer with disease progression following at least one line of endocrine therapy (61).

In addition to SERDs and PROTACs, complete estrogen receptor antagonists have also attracted increasing attention (62, 63). CERANs, represented by OP-1250, can not only inhibit ER activity but also simultaneously block the two transcriptional activation domains, AF1 and AF2, while promoting ER degradation. As a result, these agents exert a strong inhibitory effect on ER signaling (64). Preclinical studies suggest that OP-1250 may penetrate the blood–brain barrier, raising the possibility that CERANs could have therapeutic potential in endocrine-resistant breast cancer with brain metastases; however, clinical evidence supporting this application remains limited (65).

### 3.2 Precision blockade strategies targeting bypass signaling pathways

For resistance driven by activation of the PI3K/AKT/mTOR pathway, drug development has gradually shifted from early broad-spectrum inhibitors toward more precise, isoform-selective agents (66, 67). Inavolisib is a representative example of this approach (68). It selectively inhibits the PI3Kα isoform and can also promote degradation of the mutant p110α protein, making it particularly relevant for patients with PIK3CA-mutant disease (69). In the phase III INAVO120 trial, the first-line triplet regimen of inavolisib plus palbociclib and fulvestrant prolonged median progression-free survival (PFS) to 15.0 months in patients with PIK3CA-mutated breast cancer, compared with 7.3 months in the control group. The objective response rates also differed markedly between the two groups, at 58% and 25%, respectively. Subsequent overall survival analysis showed that the median overall survival (OS) reached 34 months in the inavolisib group, compared with 27 months in the control group, with an updated PFS hazard ratio of 0.42 (70). Compared with earlier PI3K inhibitors, inavolisib appears to offer a more manageable safety profile, with adverse events that are generally considered monitorable and clinically manageable. For patients with PIK3CA-mutated disease who have a high tumor burden or poor prognostic features, this regimen provides a new therapeutic option.

At the level of the AKT pathway, capivasertib is currently one of the most representative pan-AKT inhibitors (71, 72). The CAPItello-291 trial showed that, in patients with HR+/HER2− breast cancer who had progressed after endocrine therapy, capivasertib combined with fulvestrant significantly improved treatment efficacy. In the overall population, median progression-free survival (PFS) was 7.2 months in the combination group, compared with 3.6 months in the control group. Among patients harboring PIK3CA, AKT1, or PTEN alterations, median PFS was 7.3 months and 3.1 months, respectively. Notably, some benefit was also observed in patients without detectable AKT pathway alterations, suggesting that AKT inhibitors may not be limited to populations with clearly defined pathway mutations. For patients with PIK3CA-mutated disease who experienced progression after CDK4/6 inhibitor therapy, the BYLieve study explored the value of alpelisib plus fulvestrant as a second-line treatment. Among 127 patients, 53.8% remained free from disease progression at 6 months after receiving this regimen, and the overall toxicity was manageable. These findings support the important role of PI3Kα inhibitors in the treatment of advanced HR+/HER2− breast cancer (73, 74). Therefore, combining endocrine agents with PI3K or AKT inhibitors in effect suppresses both the primary ER-driven pathway and bypass growth signaling (75). For resistance driven by activation of the PI3K/AKT pathway and enhanced signaling crosstalk, such combination strategies have become an important clinical approach.

mTOR pathway inhibition is also an important strategy for overcoming endocrine resistance. In the BOLERO-2 trial, the mTORC1 inhibitor everolimus combined with exemestane prolonged progression-free survival (PFS). Median PFS increased from 2.8 months to 6.9 months by local assessment and from 4.1 months to 10.6 months by central assessment. However, this regimen was also associated with adverse events such as grade 3–4 stomatitis, infection, rash, and hyperglycemia, indicating that its clinical use requires careful balancing of efficacy and toxicity (76). To overcome feedback activation induced by mTORC1 inhibition, subsequent studies have focused on dual mTORC1/2 inhibitors (77). In a phase II study, sapanisertib plus fulvestrant prolonged median PFS from 3.5 months to 7.2 months, although the overall survival benefit was not evident, and toxicities such as gastrointestinal reactions and hyperglycemia limited its clinical application. The TRINITI-1 trial explored the triplet regimen of ribociclib, everolimus, and exemestane, which achieved a median PFS of 5.7 months, suggesting that some patients may benefit, although the duration of efficacy remained limited (78). In recent years, the multitargeted inhibitor gedatolisib has attracted increasing attention. It can simultaneously target multiple PI3K isoforms as well as mTORC1/2 (79, 80). The phase III VIKTORIA-1 trial showed that, in patients with PIK3CA wild-type disease who had previously received CDK4/6 inhibitors, gedatolisib combined with palbociclib and fulvestrant increased median PFS to 9.3 months, compared with 2.0 months in the control group, with relatively low rates of hyperglycemia and stomatitis. Overall, mTORC1 inhibition alone is effective but limited by feedback activation and toxicity. Future treatment strategies may increasingly shift toward multi-node blockade of the PI3K/AKT/mTOR pathway, combined with endocrine therapy or CDK4/6 inhibitors, to achieve more durable therapeutic benefit.

Strategies after CDK4/6 inhibitor progression. The value of continuing or switching CDK4/6 inhibition after progression is not uniform across trials (81). In MAINTAIN, switching endocrine therapy and generally switching the CDK4/6 inhibitor to ribociclib improved median PFS to 5.29 versus 2.76 months (HR, 0.57) (82). By contrast, continuation or rechallenge with palbociclib did not significantly improve PFS in PACE (4.6 vs. 4.8 months; HR, 1.11) or PALMIRA (4.9 vs. 3.6 months; HR, 0.84; P = 0.149) (83, 84). In the phase III postMONARCH trial, abemaciclib plus fulvestrant produced a statistically significant but modest investigator-assessed PFS improvement over fulvestrant alone (6.0 vs. 5.3 months; HR, 0.73), with stronger separation by blinded central review (85). Together, these results do not support indiscriminate continuation of the same CDK4/6 inhibitor; treatment selection should consider the endocrine partner, whether the CDK4/6 agent is changed, prior duration of benefit, resistance biomarkers, disease tempo, toxicity, and access to biomarker-directed alternatives.

### 3.3 Novel combination therapies and epigenetic interventions

In addition to directly targeting signaling pathways such as PI3K/AKT/mTOR, epigenetic modification and metabolic reprogramming have gradually emerged as new directions in the study of endocrine resistance. Given the overexpression of EZH2 in resistant cells and its role in maintaining ER transcriptional activity, the combination of EZH2 inhibitors with endocrine therapy is currently undergoing early clinical evaluation (14). EZH2 inhibitors represented by tazemetostat are mainly intended to correct the aberrant epigenetic state of resistant cells, thereby restoring their sensitivity to endocrine therapy (86). On the other hand, HER2/HER3 signaling can influence the acetylation status of FOXA1 through HDAC2, further altering the genomic binding sites of ER on chromatin. This finding suggests that combining HDAC inhibitors with SERDs may have a rational biological basis. For example, the HER2/HER3–HDAC2–FOXA1 axis provides a biological rationale for exploring HDAC inhibitors in combination with ER-targeted therapies; however, whether agents such as romidepsin can effectively prevent resistance-associated ER transcriptional reprogramming requires further experimental and clinical validation (24).

Resistant cells also undergo metabolic adaptation, including increased dependence on fatty acid oxidation (FAO) and oxidative phosphorylation (OXPHOS) (87–89). Mechanistically, CPT1A-mediated transport of long-chain fatty acids into mitochondria supports beta-oxidation, generating acetyl-CoA and reducing equivalents that sustain mitochondrial ATP production. Therapy-tolerant persister cells may use this mitochondrial flexibility to preserve redox balance and survive estrogen deprivation in a slow-cycling state, from which resistant clones can later re-expand. FDXR-CPT1A signaling has been linked to this FAO-dependent phenotype, while enhanced OXPHOS provides an additional survival route when ER-driven anabolic programs are suppressed. In preclinical models, the CPT1 inhibitor etomoxir showed greater activity in resistant than in endocrine-sensitive parental cells and enhanced the antiproliferative effect of fulvestrant. These findings establish a biological rationale for metabolic combinations, but FAO/OXPHOS-directed treatments remain investigational, and the selectivity and tolerability of available inhibitors require further validation.

Antibody–drug conjugates (ADCs) should be distinguished from the resistance mechanisms described above. HER2-low or HER2-ultralow expression and TROP2 are therapeutic selection features or drug targets rather than direct causes of endocrine resistance. After endocrine resistance has developed, T-DXd may be considered according to HER2 expression and line-specific eligibility, whereas the TROP2-directed ADCs sacituzumab govitecan and Dato-DXd provide later-line options under their respective indications (90, 91). In TROPiCS-02, sacituzumab govitecan improved PFS and OS compared with physician’s-choice chemotherapy in heavily pretreated, endocrine-resistant HR+/HER2− metastatic breast cancer (92); a subsequent systematic review and meta-analysis supported its activity while emphasizing heterogeneity and the need to refine treatment sequencing (93). Dato-DXd was approved in the United States in January 2025 for adults with unresectable or metastatic HR+/HER2− breast cancer previously treated with endocrine-based therapy and chemotherapy for unresectable or metastatic disease (94, 95). Thus, ADCs are best discussed as antigen-directed post-resistance therapies rather than as direct resistance mechanisms. These pathway-drug relationships and mechanisms of action are summarized in **Figure 3**.

**Figure 3:**
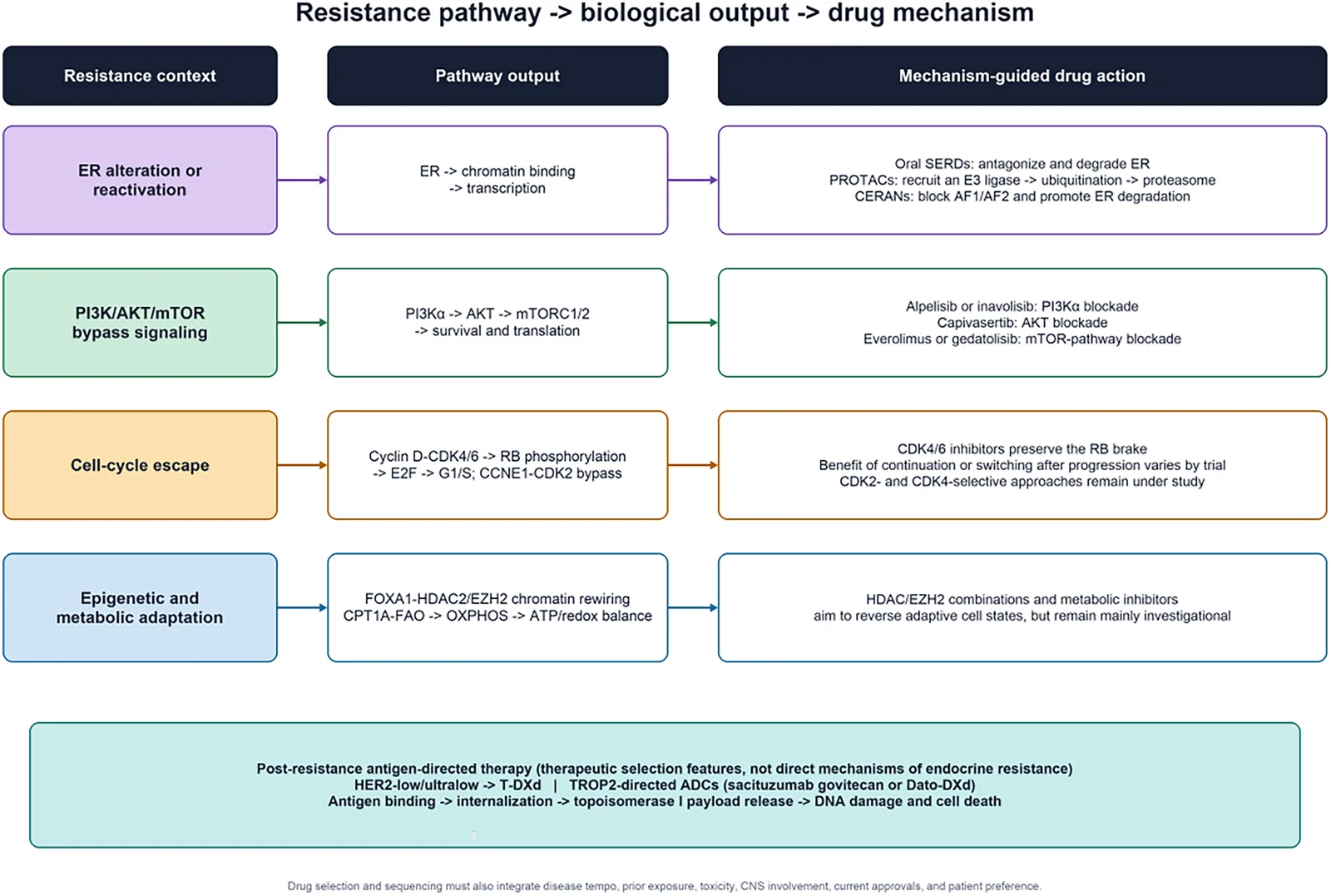
Mechanism-guided therapeutic strategies and mechanisms of action of emerging therapies for endocrine-resistant HR+/HER2− breast cancer. HER2-low/ultralow expression and TROP2 are shown as treatment-selection features or drug targets for post-resistance ADC therapy, not as direct mechanisms of endocrine resistance.

## 4 Conclusion

This review indicates that endocrine resistance in HR+/HER2− breast cancer involves multiple mechanisms, including alterations in the receptor itself, activation of bypass signaling pathways, dysregulation of cell-cycle control, and epigenetic and metabolic adaptation. These mechanisms may coexist at different disease stages or even within the same patient, making the process of resistance highly complex. Mechanistic vulnerabilities support oral SERDs, PROTACs, CERANs, PI3K/AKT/mTOR inhibitors, epigenetic agents, and metabolic modulators; separately, ADCs provide antigen-directed options after endocrine resistance has developed. The application of liquid biopsy technologies, such as ctDNA analysis, allows resistance-associated alterations, including ESR1 mutations, to be detected earlier and may help guide treatment adjustment before overt disease progression occurs. However, several issues remain unresolved. Endocrine resistance is markedly heterogeneous, and a single biomarker is unlikely to accurately reflect the full resistance landscape. The long-term efficacy, safety, optimal combination strategies, and treatment sequencing of novel agents still require further validation in clinical studies. Future research should integrate multi-omics analysis, single-cell sequencing, and dynamic monitoring to clarify the evolutionary trajectory of resistant clones. Treatment strategies should also be tailored according to each patient’s molecular features and prior treatment history. Only by integrating mechanism-based identification, real-time monitoring, and timely therapeutic adjustment can endocrine therapy for HR+/HER2− breast cancer gradually move toward more precise, whole-course disease management. A pragmatic post-resistance treatment framework follows. A proposed mechanism-guided treatment decision-making framework is illustrated in **Figure 4**.

**Figure 4:**
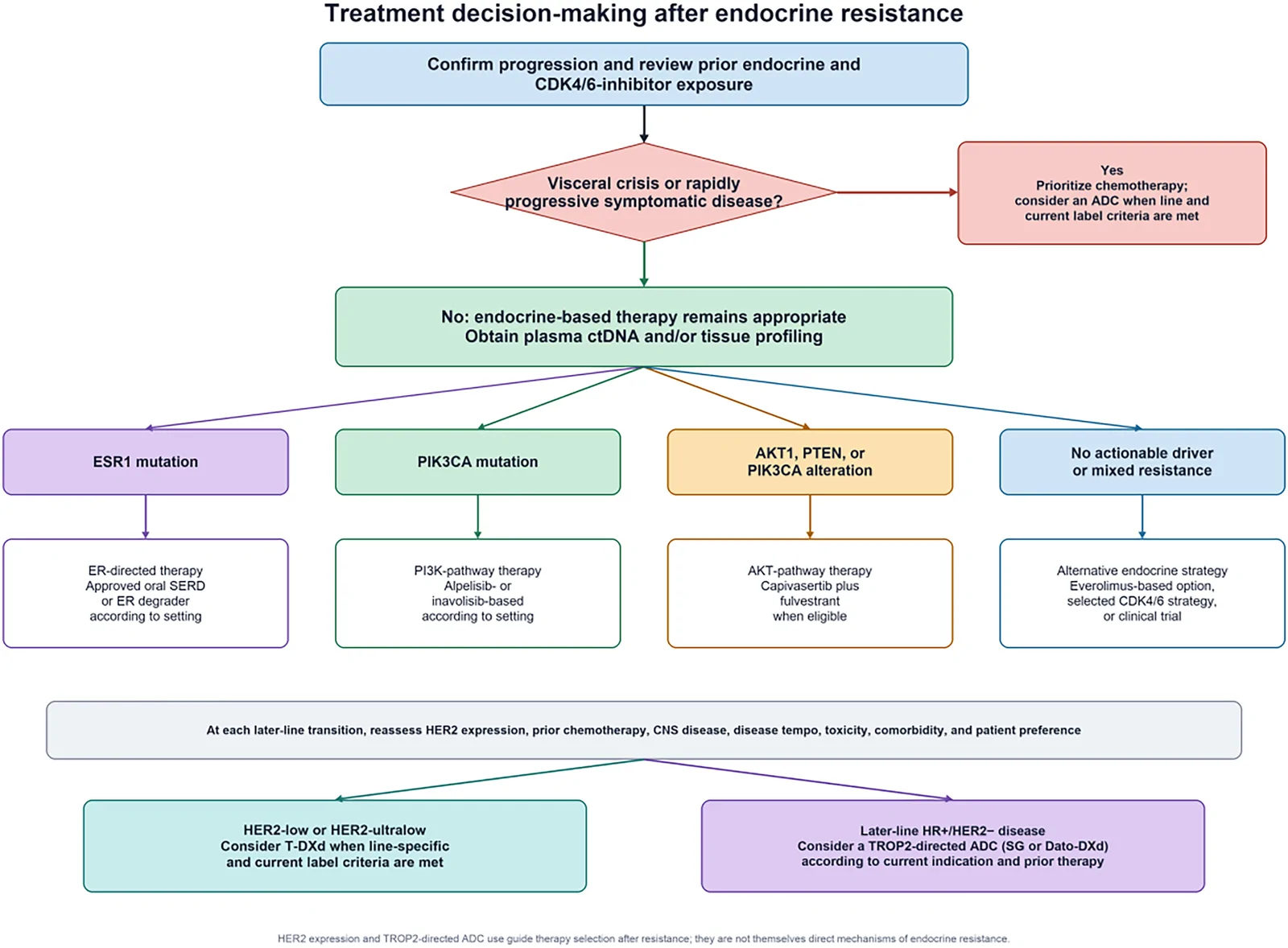
Treatment decision-making flowchart for HR+/HER2− breast cancer after endocrine resistance. Treatment sequence should be individualized according to disease tempo, prior exposure, ESR1, PIK3CA, AKT1/PTEN and HER2 status, toxicity, and the current regulatory and guideline context.

Key published or approved studies and selected ongoing studies evaluating emerging therapeutic strategies for HR+/HER2− breast cancer are summarized separately in **Tables 2**, **3**.

**Table 2:** Key published or approved clinical studies in HR+/HER2− breast cancer.

Trial	Phase/population	Treatment	Key efficacy outcomes	Status/key clinical implication
EMBER-3 (NCT04975308)	Phase III; HR+/HER2− advanced/metastatic breast cancer after ≥1 prior line of endocrine therapy; includes an ESR1-mutated subgroup	(1) Imlunestrant monotherapy vs standard endocrine therapy (fulvestrant or exemestane); (2) imlunestrant + abemaciclib vs imlunestrant	ESR1-mutated: median PFS 5.5 vs 3.8 months; updated combination analysis: median PFS 10.9 vs 5.5 months (HR 0.59); ESR1-mutated median OS 34.5 vs 23.1 months (HR 0.60; significance boundary not reached)	FDA approved imlunestrant on Sep 25, 2025 for ER+/HER2−, ESR1-mutated advanced/metastatic disease after ≥1 line of endocrine therapy; updated efficacy: Ann Oncol. 2026;37(4):532–543
SERENA-6 (NCT04964934)	Phase III; HR+/HER2− advanced breast cancer receiving first-line AI + CDK4/6 inhibitor; emergent ESR1 mutation detected by ctDNA before radiographic progression	Early switch to camizestrant + the same CDK4/6 inhibitor vs continuation of AI + CDK4/6 inhibitor	Updated median PFS 16.8 vs 9.2 months (HR 0.45); median PFS2 25.7 vs 19.1 months (HR 0.63)	Supports ctDNA-guided early treatment switching after emergence of ESR1 mutation and before radiographic progression; extended analysis: Lancet Oncol. 2026;27(8):941–958
VERITAC-2 (NCT05654623)	Phase III; ER+/HER2− advanced breast cancer previously treated with a CDK4/6 inhibitor and endocrine therapy	Vepdegestrant (PROTAC ER degrader) vs fulvestrant	ESR1-mutated cohort: median PFS 5.0 vs 2.1 months (HR 0.58); overall population: 3.8 vs 3.6 months (HR 0.83; P = 0.07), not statistically significant	PFS benefit was concentrated in the ESR1-mutated population; final phase III results: N Engl J Med. 2025;393(6):556–568
VIKTORIA-1 (NCT05501886)	Phase III; HR+/HER2−, PIK3CA wild-type advanced breast cancer after progression on CDK4/6 inhibitor + AI therapy	(1) Gedatolisib + palbociclib + fulvestrant vs fulvestrant; (2) gedatolisib + fulvestrant vs fulvestrant	Triplet: median PFS 9.3 vs 2.0 months (HR 0.24); doublet: median PFS 7.4 vs 2.0 months (HR 0.33)	Pan-PI3K/mTORC1/2 inhibitor; FDA approved gedatolisib + fulvestrant, with or without palbociclib, on Jul 14, 2026 for HR+/HER2− locally advanced/metastatic disease without a detected PIK3CA mutation after progression on or after ≥1 endocrine-therapy line in the metastatic setting
INAVO120 (OS update) (NCT04191499)	Phase III; PIK3CA-mutated HR+/HER2− advanced breast cancer in the first-line setting	Inavolisib + palbociclib + fulvestrant vs placebo + palbociclib + fulvestrant	Median PFS 15.0 vs 7.3 months (HR 0.42); median OS 34.0 vs 27.0 months	Selective PI3Kα inhibitor with mutant p110α degradation activity N Engl J Med. 2024;391(17):1584–1596; N Engl J Med. 2025;393(2):151–161
CAPItello-291	Phase III; HR+/HER2− advanced breast cancer with progression during or after endocrine therapy (n = 708)	Capivasertib + fulvestrant vs placebo + fulvestrant	Overall population: median PFS 7.2 vs 3.6 months; AKT-pathway-altered population: 7.3 vs 3.1 months	Pan-AKT inhibitor; benefit was greatest in tumors with PIK3CA/AKT1/PTEN alterations N Engl J Med. 2023;388(22):2058–2070
TRINITI-1	Phase I/II; HR+/HER2− advanced breast cancer after progression on a CDK4/6 inhibitor	Ribociclib + everolimus + exemestane	Median PFS 5.7 months	Triplet strategy after CDK4/6 inhibitor progression; activity was modest Clin Cancer Res. 2021;27(15):4177–4185

**Table 3:** Selected ongoing or actively developing clinical studies in HR+/HER2− breast cancer.

Trial	Phase/population	Treatment	Key efficacy outcomes	Status/key clinical implication
lidERA BC (NCT04961996)	Phase III (adjuvant); Intermediate-/high-risk stage I–III HR+/HER2− early breast cancer (n = 4,170)	Giredestrant vs standard endocrine therapy (AI or tamoxifen)	iDFS HR 0.70; 3-year iDFS 92.4% vs 89.6%; premenopausal HR 0.65 and postmenopausal HR 0.74	Reported as the first oral SERD to show benefit in the adjuvant setting; regulatory review ongoing at the time of the cited report SABCS 2025 presentation; Roche press release
ELEVATE (NCT05563220)	Phase Ib/II umbrella study; HR+/HER2− advanced breast cancer; ESR1 mutation not required	Elacestrant combined with everolimus, abemaciclib, or capivasertib	Elacestrant + everolimus: median PFS 8.3 months, DCR 82.9%; + abemaciclib: 14.3 months, DCR 91.2%; + capivasertib: 11.3 months	Combination activity reported across ESR1 mutation subgroups; early-phase data ASCO 2025 abstract PS7-06
FOURLIGHT-1 (NCT06105632)	Phase II; Disease progression after prior CDK4/6 inhibitor therapy; heavily pretreated population	Selective CDK4 inhibitor atirmociclib + fulvestrant vs standard therapy	PFS HR 0.60; 6.4% discontinued because of adverse events	Selective CDK4 inhibition is being explored to preserve cell-cycle blockade while potentially reducing CDK6-related myelosuppression; phase III development ongoing Pfizer press release, Mar 17, 2026
